# Migration as a risk and a livelihood strategy: HIV across the life course of migrant families in India

**DOI:** 10.1080/17441692.2016.1155635

**Published:** 2016-03-22

**Authors:** Tanvi Rai, Helen S. Lambert, Helen Ward

**Affiliations:** ^a^ School of Public Health, Imperial College London, London, UK; ^b^ School of Social and Community Medicine, University of Bristol, Bristol, UK

**Keywords:** Migration, HIV, qualitative, India, livelihood

## Abstract

Migrant workers are understood to be vulnerable to HIV. However, little is known about the experience of migration-based households following HIV infection. This qualitative study examined the migration-HIV relationship beyond the point of infection, looking at how it affects livelihood choices, household relationships and the economic viability of migrant families. We conducted semi-structured interviews with 33 HIV-positive migrant men and women recruited from an anti-retroviral therapy (ART) centre in north India. Following infection among the migrant men, contact with free, public-sector HIV services was often made late, after the development of debilitating symptoms, abandonment of migrant work and return to native villages. After enrolment at the ART centre participants’ health eventually stabilised but they now faced serious economic debt, an inflexible treatment regimen and reduced physical strength. Insecure migrant job markets, monthly drug collection and discriminatory employment policies impeded future migration plans. HIV-positive wives of migrants occupied an insecure position in the rural marital household that depended on their husbands’ health and presence of children. The migration-HIV relationship continued to shape the life course of migrant families beyond the point of infection, often exposing them again to the economic insecurity that migration had helped to overcome, threatening their long-term survival.

## Introduction

In most of the developed world, the availability of effective anti-retroviral therapy (ART) has transformed the experience of living with HIV, rendering it similar to other chronic diseases (Gifford & Groessl, 2002; Mitchell & Linsk, 2004; Swendeman, Ingram, & Rotheram-Borus, 2009). However, in resource-limited countries, social and economic circumstances make the management of HIV difficult (Marsland, 2012; Prince, 2012) and managing HIV as a chronic condition poses additional challenges in the pursuit of sustainable livelihoods (Russell et al., 2007). A number of studies, primarily based in sub-Saharan Africa, have documented the impact of HIV on affected households and family relationships. The high risk of HIV infection among young adults, most likely the primary income earners or carers of young children, adversely affects the entire household, increasing families’ vulnerability to premature death, chronic morbidity and economic impoverishment (Bachmann & Booysen, 2003). This may lead to household dissolution and migration of some family members to relieve financial pressure (Hosegood, McGrath, Herbst, & Timaeus, 2004; Hosegood, Preston-Whyte, Busza, Moitse, & Timaeus, 2007; Seeley et al., 2008; Tekola, Reniers, Haile Mariam, Araya, & Davey, 2008; Yamano & Jayne, 2004). The effects of HIV on people's lives are cumulative; once households are HIV-affected, the effects of subsequent negative events are compounded by the prior presence of HIV. If adults become sick or die, the loss of their income (from salaries or monthly pensions), combined with costs for healthcare and funerals can cause some families to suffer food insecurity or inability to keep children in school. Gaining access to government support grants takes time and resources, both of which may not be available to households with only one or two adults and several dependents. Further, the stigma associated with HIV, sometimes exacerbated when accompanied by visible household poverty, prevents neighbours and relatives from offering practical or financial help (Bachmann & Booysen, 2003; Hosegood et al., 2007).

In contrast to the generalised epidemic in sub-Saharan Africa, in India population-level HIV prevalence is low and infection is concentrated among high-risk groups such as sex workers and injecting drug users (NACO, 2013). While there are fewer published studies from India, they are similar accounts of the negative impact of HIV and HIV-related stigma on employment and assets, the exacerbation of existing gender inequalities and social isolation of HIV-affected families (Bharat & Aggleton, 1999; Pallikadavath, Garda, Apte, Freedman, & Stones, 2005; Taraphdar et al., 2011).

Another group that is of increasing interest to health programmers in India are circular migrant workers who migrate for a few months a year to pursue paid employment. There is international evidence of an association between labour migration and higher levels of sexual-risk behaviour and HIV infection (Campbell, 1997; Gupta & Singh, 2002; Mercer, Khanam, Gurley, & Azim, 2007; Ondimu, 2010; Pison, Leguenno, Lagarde, Enel, & Seck, 1993; Saggurti, Schensul, & Verma, 2009; Yang, 2010). In India, of greatest concern are rural-to-urban migrant workers who may act as a ‘bridge’ for HIV to travel from higher prevalence cities to low-prevalence rural areas (Chandrasekaran et al., 2006; NACO, 2010, 2013; Rai et al., 2014). However, to our knowledge, the impact of HIV infection on these already vulnerable communities and families has not been explored.

In this study we explore the effects of HIV infection on the life course of circular migrant workers in India, examining how the interaction between migration and HIV continues to affect people after infection, diagnosis and starting care. Based on a qualitative study set in an ART centre in a high-outmigration district in Uttar Pradesh state, we document the main issues confronting those trying to manage life with HIV, and the consequences of HIV in their life course.

## Methods

### Study setting

The study was carried out in Allahabad, one of five high HIV prevalence districts within the north Indian state of Uttar Pradesh (UP), which has a low-prevalence overall. This paper presents findings from one component of a multi-site qualitative study exploring the relationship between migration and HIV which included observations and interviews in two rural locations with key informants and migrant families, and in one clinic setting, the Allahabad district ART centre where we interviewed staff and patients. Data from this clinical setting are analysed for this paper. Patients come to the centre from across Allahabad district, as well as from neighbouring districts where there are no (or limited) ART services. The ART centre is located within a major district-level hospital attached to a medical college and has a daily traffic of over 100 patients, including newly diagnosed patients referred from Integrated Counselling and Testing Centres (ICTC), patients coming to collect their monthly medication, and those who develop complications. ART staff described the user profile of these free HIV public-sector facilities as being heavily skewed towards the lower socioeconomic sections of society (manual labourers, petty shopkeepers, migrant workers and truck drivers), as wealthier patients would seek treatment privately.

### Study design and sample

From December 2010 to March 2011 the first author conducted 33 semi-structured face-to-face interviews with HIV-positive men and women who had a past or continuing history of rural-to-urban migration. Most interviews took place in a private room within the ART centre. On each visit to the ART centre, patients in the waiting room were administered a brief 10-minute socio-demographic questionnaire. Those with a migration history (either themselves or via their spouse) were then invited for an in-depth interview while they were waiting for their appointments. The questionnaire also helped to purposively recruit a maximum variation sample of participants in terms of demographic and HIV-related characteristics (age, gender, time since HIV diagnosis, and whether or not they were on ARV medication). A few interviews were conducted in related clinical or community settings.

Interviews were conducted in Hindi and audio-recorded. The topic guide contained open-ended questions designed to elicit participants’ accounts of their lives and experiences as migrant workers (or their partners) before and after HIV infection. Participants were asked to recall their experiences chronologically, punctuated by significant events or stages in their lives such as marriages and other household changes, episodes of illness, various migrant jobs and their experience of migrant life, trips home, HIV diagnosis, HIV-related deaths, making first contact with the ART centre, starting treatment, etc.

### Ethical procedures

Ethical clearance was obtained from Imperial College Research Ethics Committee and from the local Institutional Ethics Review Board based at the Population Resource and Research Centre, Allahabad, India. Before each interview, participants were provided information about the research and invited to ask questions and signed a consent form before taking part in the study. If they were unable to read and write, consent was taken orally in the presence of a witness, who signed on their behalf.

### Data management and analysis

Interviews were translated and transcribed into English prior to analysis. Data were analysed using Framework (Pope, Ziebland, & Mays, 2000; Ritchie & Lewis, 2003; Ritchie & Spencer, 1993) and thematic content analysis (Green & Thorogood, 2009), using NVIVO v9 software to store and manage the data.

## Findings

Twenty men and 13 women with HIV aged between 24 and 45 years, including two couples, were interviewed. Eighteen men were married and two were widowers; seven women were married, five were widows and one was separated. In-keeping with local patterns of circular labour migration (de Haan & Rogaly, 2002; Srivastava, 2005), it was men who migrated for work while women were wives (or widows) of migrant men. Two women had accompanied their husbands to their migration destination; seven had remained with the husband's family in the village throughout, while four had spent some time at migrant destination after marriage but returned, coinciding with the arrival of children. Men reported migrating to Mumbai, Delhi, Gujarat and Punjab to work in factories, transport, construction or trading. Most participants had been diagnosed with HIV for more than six months, and two-thirds were on ART while the other nine (five men, four women) attended every six months to see whether they were now eligible for treatment.

We identified a set of complex interactions between HIV and migration, which go beyond the standard epidemiological understanding of migration as a risk factor for HIV. Looking across the adult life course of circular migrants and their families, we describe key mechanisms by which migration and HIV interact to mould their future livelihoods.

### Migration and diagnosis

Most study participants (or their husbands, in case of women) had stopped migrating for work in the lead up to their HIV diagnosis. For a few this was a coincidence – they had planned to migrate for only a short period and their episode of illness and/or HIV diagnosis had simply hastened their original intention to return home. This pattern was found among interviewees with assets such as land holdings or successful businesses, who belonged to supportive and resourceful kin networks, or who already had adult sons to support them financially. One man, following his diagnosis, was able to return to a reasonable pre-migration occupation (as a local priest performing simple religious ceremonies) as a result of his high caste status. However, the majority of participants owned little or no land and lacked strong social networks in their home village. They had invested in migration as their primary means of securing a livelihood and future for themselves and their families and were only part-way through their intended period of migrant work. They had planned to continue migrant work until they had accumulated some savings, built a house to retire in, paid off loans, married off their daughters, and their sons would be old enough to start earning. Despite the variability within the migration experience, the longer the time they spent doing migrant work, the greater was the expectation of safeguarding a future for the family.

The acquisition of HIV thus disrupted such plans for many men who stopped migrating due to illness. They described first attending the ART centre after a lengthy journey that had included some combination of prolonged physical suffering, repeated misdiagnoses, expensive privately-obtained medical treatments, financial crises and an often-hasty abandonment of previously established livelihood engagements (living arrangements and income-generating activities) (Rai, Lambert, & Ward, 2015).

… Nobody working. And whatever savings there were, they all got finished. In fact, even the land that was in our share, we had to sell it, in order to buy the medicines. It cost around 150--200 thousand rupees for medicines. And if you got to a private nursing home, they dominate and exploit you, and we just wouldn't get any clarity about what was wrong. Then with those medicines, things would get better for a bit, but then again it would be same. (Clinic-6)

With HIV having disrupted migration in this way, those who subsequently started treatment talked of it as no less than miraculous as they began to feel better, gained weight and found themselves able to function again. Participants appeared grateful for the testing and treatment received from the centre and were keen to sustain improvements in health by closely following instructions regarding health maintenance, such as diet and pill regimens.

### Living with HIV

Gaining access to free ART meant that migrant men who had become sick were no longer dying, but they were not healthy either. Although they felt better on treatment, they still had episodes of illness, for which they had been grateful to be at home in the care of their family.

I'm not able to plan to go away yet. I just feel a bit nervous because say, I've got this disease, what if it turns serious? If I'm here then at least ( … ) there's my father, brother, sister-in-law. So I'm carrying on with everybody's support. (Clinic-23)

As their health stabilised the responsibility of providing economic stability to the household gradually returned to them. Heightening this social expectation was the fact that many families now faced significant financial debts accumulated over the period of illness and unemployment prior to making contact with the ART centre. However, the need and desire to stick to ART treatment and HIV management recommendations was now impacting in a major way on participants’ future possibilities for work and migration. For example, the organisation of ART treatment delivery was a significant structural factor influencing migration decisions: patients have to collect drugs in person every month from the ART centre which is only open during regular working hours and not in the evenings (NACO, 2007, 2008). Although there is a process by which patients can transfer their registration to an ART centre to a different city, this is of limited value in the case of circular labour migrants, as it fails to accommodate the erratic and unstable nature of migrant work.

If I get it [my registration at an ART centre] transferred and I go in a month, then where will I get a job, and how will I get transferred in a month, and will I or won't I get a job. Who can be sure? … (Clinic-32)

The above participant worried that no migrant employment he could find would be secure enough to risk transferring his ART registration to that location; he also felt that the monthly visits to the centre left him insufficient time to even find suitable employment. Even asymptomatic participants not yet on treatment had to incorporate six-monthly trips to the ART centre on their visits home, but this was more manageable and they felt relieved their HIV was still at a stage where they could plan for the future.

The uncertainties of living with HIV – faltering health and the practical issues regarding accessing care – were loaded on to the already-present insecurity of migrant work-based livelihoods. Most of the migrants interviewed worked in unskilled or semi-skilled jobs – their physical bodies were all they had to offer in return for a livelihood and HIV had attacked and weakened that very asset. Most did not hold a permanent position at their places of work and were hired on a casual basis, via a contractor. They were paid for their daily attendance at work (or sometimes per piece produced) and were not usually entitled to sick pay. Self-employed workers, such as taxi drivers, only earned money when they worked but would still have to pay for hiring the vehicle (unless they owned it).

I'll go back there, back to Delhi. I'll have to look for work over there. I don't have enough money to set up my own business … I'll go to some factory … and I'll do it for a month, or two months … Now I have to come here every month. They don't give enough medicines for two months or three months … they only give it for a month. ( … ) You can get a transfer, but only after one finds a good job over there, only then would one consider living there. Only then would it make sense to get a transfer. (Clinic-19)

In addition, men were apprehensive that their reduced strength and increased vulnerability to sudden illness compromised their ability to find and retain a job. Working as a migrant seemed incompatible with the medical advice to take their medicines on time, eat healthy food at regular mealtimes and boil their drinking water, given their long shifts at work and poor living arrangements. Thus, the structural realities of working as a migrant created both everyday and longer term concerns about how to manage their HIV.

The psychological and physical impact of HIV in limiting migration opportunities was exacerbated by anticipated and enacted HIV stigma and discrimination for some men. A couple of men were prevented from taking up jobs in Gulf states as a results of direct discrimination against people with HIV, and those still in jobs feared dismissal if their HIV status was revealed. The following respondent had been diagnosed with HIV the day before being interviewed, as part of routine pre-operative screening (surgery):

I*:* ‘And when you go back, back to the company, then will you tell people there?’

R: ‘No.( … ) Over there, if the [Employer name] company finds out, then they'll throw me out … . no, they will throw me out … ’ (Clinic-7)

Men looked for ways to protect themselves from HIV-related stigma, even if it entailed compromising their social or economic status. One man had been sacked by his company after 15 years of employment following his HIV discovery. After spending two years of sickness back at home, he was now starting to explore a new job possibility in a different location, but the job was at a much lower position than he had previously. He planned to keep his HIV status secret and to work for a year before he considered transferring his ART registration:

… It's a [private] limited factory, it's not a government factory. It's casual labour work. If I were to say to you, ‘Please transfer me’. And when I get there they might say, ‘There isn't any work right now.’ ( … ).. there are no guarantees. … .Right? And so they might send me back, or not employ me. Then I'll come back to … [the ART clinic], and then they'll say, ‘Why do you keep hassling us?’ (Clinic-22)

Although respondents spoke of the ART centre as a godsend, they never assumed any personal right or entitlement to its services. Here they received expensive tests and medications for free, which was unlike their usual medical encounters. Similar to other Indian medical settings (Datye et al., 2006; Fochsen, Deshpande, & Thorson, 2006), they were keen to please clinic staff, avoiding anything that they feared could jeopardise their access to ART. For the above respondent, this included compromising his employment options simply to remain in favour with those believed to be the gatekeepers of his free treatment.

In their home villages, further structural factors were at play. Study participants felt dismay at the lack of local opportunities for employment. Rural-based jobs tend to be poorly remunerated and labour-intensive and their reduced strength constrained their options. Adding to these restrictions were those based on caste affiliations and household status within the village limiting access to certain jobs. A lack of leverage with powerful local elites in the village also affected access to financial support, including jobs, loans and even government-sponsored social protection schemes. The latter are meant for financially-poor families and rely on eligible families possessing a ‘ration’ card proving identity and local residence (Abbas & Varma, 2014; Deshingkar & Akter, 2009), but local officials issuing these cards may expect a bribe in return.

People say to me, ‘You can get a [ration] card made. And all your problems will be solved. Your medical expenses will become free.’ But nothing comes of that. If you give them money, then the card will be made. One needs to pay money for a bribe first. But I don't have money … (Clinic-15)

A new scheme for a universal biometric identity card is currently being rolled out in some states and is eventually intended to be issued to all citizens over 18 years, but some practical issues in its application to migrant workers are yet to be resolved (Abbas & Varma, 2014; Faetanini & Tankha, 2013).

### The impact on households

Men had adopted migrant work as a livelihood strategy in order to strengthen their multi-generational household, with normatively-defined roles in place for themselves as primary earners and their wives as primary carers for their children and (the man's) parents. However, when HIV-related illness struck and migration-based remittances stopped, men, and sometimes their wives as well (if they became too unwell to carry out domestic chores), became an overall burden on the household rather than an asset.

Men's concerns about their fragile health created a tension between individual needs (wanting to stay at home to be cared for by family) and household needs (inter-generational family responsibilities and expectations that needed financial investment), each having opposite effects on the likelihood of future migration:

No, right now madam, I don't have any plans to go anywhere, because my health isn't stable and, even though I do really feel like going away [migrate for work], and earn in an unconstrained way, I do have three girls and I have to marry them off ( … ) one needs 80 thousand to 1 million rupees, and then, so when I think about all these things in the future, then I will have to go, but, because currently my health is so unstable so that's why what could I do even if I went. (Clinic-12)

Living with HIV had altered family relationships and dependencies. Men had often become financially dependent on fathers, brothers or sons depending on their age at diagnosis and what stage they had been in the life course. More than half the men/husbands had been living at home for the past six months to a few years. For many, the medical crises that had resulted in their return from migrant locations had ultimately evolved into a chronic illness. Correspondingly, family members had come to the rescue when things were critical, but as illness stabilised, that pure concern was gradually replaced by a grudging charity. Although the migrant man and wife helped out in local income-generating activities when they were feeling strong, for instance in agricultural jobs or running a family shop, this was only enough to meet their basic daily consumption needs. Furthermore, in families where migrant work had spanned several generations, other earning members such as brothers or cousins were often migrants themselves, based outside the village, who preferred to send remittances to their own (marital) families rather than support their siblings.

One woman talked about how her father-in-law had recalled them from Mumbai when her husband's health started to fail. They believed it to be the right decision at the time:

It used to be casual work, you only get money for the days you work. If you don't do it then there is nothing. So having thought about all this, we thought, ‘Let's go live at home, in fact that is better. At least we'll get a meal once a day then’. (Clinic-31, wife of migrant)

However, after nine months of being back at the marital home, relations had started to sour.

No, [we] don't have the courage to go back. How can we ask [for the money]? ( … ) When we're not earning anything then … when we ask him for money to get around then he gives us quite an earful. ‘How many persons’ expenses am I supposed to take care of? How many people am I to be responsible for?’ But we are also left with no choice. If we were well then we could live and work anywhere. (Clinic-31)

For married women, becoming HIV-positive compounded the negative effects of their already low status in the marital household and had profound consequences for whether they remained in their marital home or returned to live with their parents. Despite the above-described problems, the man's status was still safeguarded in the family unit but the woman's ability to retain her position was based on the continuation of her role as a caring wife, daughter-in-law, and mother of dependent children.

Half of the HIV-positive women were either widowed or separated from their migrant husbands. The other half were living with their husbands and/or husbands’ family (if husband still migrating). For the six interviewees who were widows or separated from their husbands, the relationship with their husbands’ family was affected by the presence of children: in general, having children, and especially sons, secured some level of their support but chronic illness or disputes over finances often led to women moving back to their natal homes.

Women who were relatively healthy and women with supportive husbands had similar concerns as the men regarding the future.

Now … it's like we've got a new life. We are living a new life. ( … ) … our children, I've asked god for just as much time, no matter how long the rest of our life is, but if we could get at least 20 more years of life, so that we can settle our youngest daughter into her life, and then after that, I pray to god, that no matter when you want, you can call me, and I'm ready. (Clinic-3, wife of ex-truck driver)

However, among the women for whom HIV had resulted in marital breakdown, or widowhood in combination with declining health, they were concerned only with the immediate fragility of their own existence. They did not see any way out of their situation and felt themselves to be a burden on their families. For instance, one young woman who had moved back with her parents after separating from her husband was becoming increasingly frail and unable to carry out daily domestic tasks. Another woman was living at her natal home waiting for her migrant husband to call her to Mumbai. She was looking after two small children with the support of her parents, but had no alternative plans in case the invitation did not come.

## Discussion

The effect of HIV on migrant households has not previously been studied in India. The findings presented here show that interaction between migration and HIV continues beyond the point of infection and influences the long-term course of lives and households. Migration may initially increase migrants’ susceptibility to HIV but following infection, this relationship is reversed and HIV starts to regulate people's ability to continue migrating. Our study participants had opted for migration as a livelihood strategy in the face of limited, poorly paid rural employment options and a lack of material and social assets. Migration helped them mitigate these disadvantages by providing an alternative means of income and prospects of longer term betterment of their domestic circumstances. However, being forced to cease migrant work as a consequence of HIV returned them to their pre-migration situation but with the added burdens of significant debt due to long periods of illness, reduced physical strength, and the demands of an inflexible new treatment regimen. Being HIV-positive thereby became an embodiment of their material disadvantage.

The effects of HIV on the life courses of migrant families operate at several levels. At individual level, male participants lamented how their chronic HIV-related weakness and morbidity had diminished employment options, adding to concerns about HIV-related prejudice and discrimination. On the spectrum of financial security following an HIV diagnosis, at one end, a minority were shielded from disaster due to higher background levels of social and/or economic advantage in their home village, but most migrant families were concentrated at various points further along, where their already fragile livelihoods were anticipated to get worse. As found elsewhere (Fitzgerald, Collumbien, & Hosegood, 2010; Siu, Wight, & Seeley, 2012), men also expressed guilt at being unable to fulfil their social roles within the household. They had expected to use their income from migration towards building better housing, getting children educated and married and providing support and care to ageing parents (de Haan, 1997; Rogaly, 1998) . Instead, they were now concerned with basic survival and the future security of their children. HIV also eroded much of the social capital that women had accumulated in their marital homes through years of performing domestic duties and bearing children (Jeffery & Jeffery, 1997). Even their continued existence in the marital home came under threat when their migrant husbands stopped being productive, became sick or died, or when they themselves became sick with HIV-related conditions.

These problems in turn were exacerbated by structural conditions including the informal organisation of migrant employment, where workers are employed on a casual basis with limited access to healthcare, no financial protection against the onset of illness and lack of working arrangements for those needing long-term care (Borhade, 2011; Deshingkar & Akter, 2009; Srivastava, 2005). Moreover the organisation of ART services, with inconvenient opening hours, long waiting times and inflexible registration transfer system, made obtaining treatment logistically incompatible with migrant work. Yet staying at home was incompatible with pursuing productive activities that HIV-positive individuals would be sufficiently healthy to do and that would provide net economic returns. Thus HIV infection and associated sickness and death did not act alone in derailing the lives of migrant families – many contextual and structural constraints combined to produce such disruption and HIV acted to expose and compound these. These effects varied depending on life stage, gender, level of debt, HIV stage and the strength of social networks at home.

This study has certain limitations. Interviews are notoriously sensitive to social desirability bias, potentially producing undue emphasis on self-reported perceptions. We therefore placed the subjective realities narrated by participants against broader understandings of context derived from a range of academic and non-academic sources. We also carried out interviews with ART clinic staff, which corroborated many of the issues mentioned by our respondents. As with longitudinal case studies (Russell, 2005; Seeley et al., 2008), a life course approach (Ben-Shlomo & Kuh, 2002) can provide insight into the relationships between exposures people face at different biographical stages and different outcomes and has recently been recommended for studying migrant health (Spallek, Zeeb, & Razum, 2011). To achieve a similar effect, we used a biographical approach in eliciting responses. However, we were restricted to series of single interviews, which may suffer from recall bias. The need to accommodate research timetables and the desire for single-contact only by respondents in a clinic environment prohibited further interviews. Finally, being interviewed in clinic settings could have introduced further bias, although we stressed the independent status of our research, and that our focus was on patients’ experiences of living with HIV and not about their clinical care.

## Conclusion

Much has been written about structural violence and HIV risk, highlighting the effects of social, political and economic inequality on vulnerability to HIV infection, so that the poor and socially marginal are rendered most vulnerable (Farmer, 1999; Parker, 2002; Rhodes et al., 2012). The circumstances that caused families in our research to choose migration as a livelihood strategy may also have simultaneously increased their vulnerability to HIV, as attested by other work on migrant workers (Mtika, 2007; Organista & Kubo, 2005; Poudel, Jimba, Okumura, Joshi, & Wakai, 2004). Our study adds to these observations by showing that the effects of structural violence continue post-infection. There were serious adverse consequences for the future life course of the entire household when a migrant head of household became sick and either died or did not recover enough to resume migration again. As found in sub-Saharan African settings (Hosegood et al., 2004; Tekola et al., 2008), some household units broke up, especially when women were widowed or ill with HIV themselves. The depletion of household savings on private medical care before contact with public-sector services was widespread in our sample and study participants described the attenuation of their children's future social and economic prospects, through inability to afford the marriage of daughters, the need for sons to assume responsibilities for supporting the family at an early age, and the stigma derived from belonging to an HIV-affected family.

Our findings are based on experiences of HIV-positive migrants who had successfully enrolled in ART services. We have not included those who have been unable to access ART, who we would expect face similar issues but even greater problems. The stigma, shame and discrimination associated with HIV continue to derail public health efforts to get people tested and into care early. The particular status of migrant workers as a highly mobile group of individuals who often lack documentary evidence of identity, residence or employment renders them further invisible to health improvement and social protection programmes (Borhade, 2011; Faetanini & Tankha, 2013).

Thus HIV had enduring effects on migrant families that went beyond individual infection and produced a downward spiral of disadvantage for future generations. The Government of India's national AIDS control strategy recently intensified its efforts towards migrant workers (NACO, 2010), instituting awareness campaigns at high out-migration rural source areas and during transit as well as at migrant destinations (NACO, 2012, 2013). However, these programmes need to extend beyond targeting new infections to providing protection and support to migrant families who are already infected. Efforts aimed only at increasing HIV awareness fail to address the contextual factors that make migrants vulnerable to HIV infection and then later prevent them from accessing and benefitting from the available HIV services. Some simple changes in the delivery of HIV services could help, for instance, ART centres could be linked electronically across the country so that patients could collect their monthly medication from whatever large city they were closest to. Food and travel coupons may help reduce patient attrition or gaps in treatment for those struggling economically. Major structural changes in migrant-employing organisations are called for, such as a nationally-authorised and mandated recognition of migrant workers’ rights so that they are entitled to sick pay or health insurance, and their jobs are protected. Providing livelihood support for migrant families affected by HIV could encourage migrants to seek testing and treatment early, and perhaps resume their employment without catastrophic losses of health and savings, as has been observed in a South African cohort study (Bor, Tanser, Newell, & Barnighausen, 2012)

There is a public health rationale as well as economic and moral grounds for such a strategy. If economic necessity impels HIV-positive migrants to continue to migrate for work, not only will they represent continuing risks for onward transmission but the suboptimal and health-compromising circumstances in which such migrants work are likely to contribute towards faster progression into the development of full-blown AIDS and an inflation in the associated cost of care.

A fast progression towards AIDS is more likely in such circumstances due to adherence failure because of the indirect costs of maintaining contact with the ART centre (travel costs, avoidance of lost income for ART visit days, migration-based jobs), poor nutritional status, toxic side-effects as a consequence of poor diet, ARV treatment failure due to not taking pills at the correct time/dosage, and switching to alternative medicine because of unpleasant side-effects of ART or the promise of a cure (Musheke, Bond, & Merten, 2012; Sahay, Reddy, & Dhayarkar, 2011; Siu et al., 2012).

In India, lax enforcement of policies meant to protect migrants, absence of culpability of industry employers of migrant labour, a weak public health system in parallel with a flourishing private health industry, the rigid nature of the ART programme, failures of rural development initiatives to bring prosperity and equity to rural areas, and the continuing low status of women have all contributed substantially to heighten the adverse effects of HIV on rural-to-urban migrant workers and their families. Without concerted action through inter-sectoral collaboration between government departments on this range of issues, migrants are likely to continue to have their livelihoods harshly undermined by HIV, despite the availability of effective treatment.

## Disclosure statement

The authors have no conflicts of interest to declare.
